# Emerging translational strategies and challenges for enhancing regulatory T cell therapy for graft-versus-host disease

**DOI:** 10.3389/fimmu.2022.926550

**Published:** 2022-07-28

**Authors:** Keli L. Hippen, Mehrdad Hefazi, Jemma H. Larson, Bruce R. Blazar

**Affiliations:** ^1^ University of Minnesota Cancer Center and the Department of Pediatrics, Division of Blood & Marrow Transplant & Cellular Therapy, Minneapolis, MN, United States; ^2^ Division of Hematology, Mayo Clinic, Rochester, MN, United States

**Keywords:** tTreg, pTreg, iTreg, CAR, GVHD

## Abstract

Allogeneic hematopoietic stem cell transplantation (allo-HSCT) is a curative therapy for many types of cancer. Genetic disparities between donor and host can result in immune-mediated attack of host tissues, known as graft versus host disease (GVHD), a major cause of morbidity and mortality following HSCT. Regulatory CD4+ T cells (Tregs) are a rare cell type crucial for immune system homeostasis, limiting the activation and differentiation of effector T cells (Teff) that are self-reactive or stimulated by foreign antigen exposure. Adoptive cell therapy (ACT) with Treg has demonstrated, first in murine models and now in patients, that prophylactic Treg infusion can also suppress GVHD. While clinical trials have demonstrated Treg reduce severe GVHD occurrence, several impediments remain, including Treg variability and practical need for individualized Treg production for each patient. Additionally, there are challenges in the use of in vitro expansion techniques and in achieving in vivo Treg persistence in context of both immune suppressive drugs and in lymphoreplete patients being treated for GVHD. This review will focus on 3 main translational approaches taken to improve the efficacy of tTreg ACT in GVHD prophylaxis and development of treatment options, following HSCT: genetic modification, manipulating TCR and cytokine signaling, and Treg production protocols. In vitro expansion for Treg ACT presents a multitude of approaches for gene modification to improve efficacy, including: antigen specificity, tissue targeting, deletion of negative regulators/exhaustion markers, resistance to immunosuppressive drugs common in GVHD treatment. Such expansion is particularly important in patients without significant lymphopenia that can drive Treg expansion, enabling a favorable Treg:Teff ratio in vivo. Several potential therapeutics have also been identified that enhance tTreg stability or persistence/expansion following ACT that target specific pathways, including: DNA/histone methylation status, TCR/co-stimulation signaling, and IL-2/STAT5 signaling. Finally, this review will discuss improvements in Treg production related to tissue source, Treg subsets, therapeutic approaches to increase Treg suppression and stability during tTreg expansion, and potential for storing large numbers of Treg from a single production run to be used as an off-the-shelf infusion product capable of treating multiple recipients.

## Introduction

**Figure 1:**
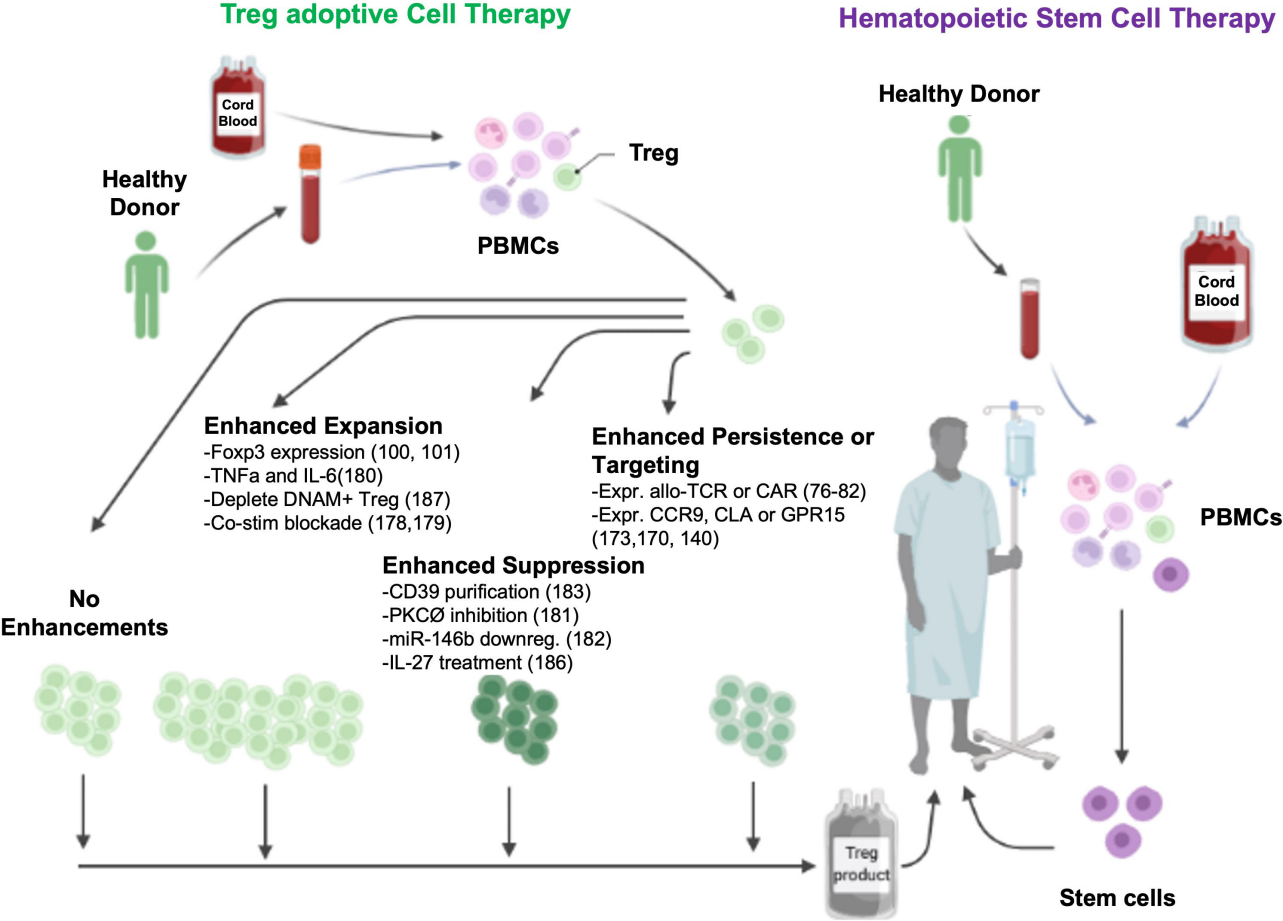
Potential ex vivo mechanisms to enhance Treg ACT.

Allogeneic hematopoietic stem cell transplantation (allo-HSCT) is a curative therapy for many types of cancer (1). Genetic disparities between donor and host can result in graft-versus-host disease (GVHD), a major cause of morbidity and mortality following allo-HSCT (2). Regulatory CD4+25++FoxP3+ T cells (Tregs) are present at low frequency and are crucial for immune system homeostasis by limiting the activation and differentiation of effector T cells (Teff) that are self-reactive or stimulated by foreign antigen (**Figure 1**) exposure (3). Adoptive cell therapy (ACT) with Tregs has demonstrated, first in murine models and now in patients, that prophylactic Treg infusion can also suppress GVHD (4–6). While clinical trials have demonstrated Tregs reduce severe GVHD occurrence, several impediments remain, including the practical need for individualized Treg production for each patient (5, 7). Additional challenges exist in achieving *in vivo* Treg persistence, especially in the context of immune suppressive drugs given to patients for GVHD prevention or treatment (8).

This review will focus on translational approaches taken to improve the efficacy of Treg ACT such as manipulating T cell receptor (TCR) and cytokine signaling, *in vitro* expansion and genome modifications to improve antigen specificity, GVHD target tissue migration, and therapeutics to enhance Treg stability or persistence/expansion following ACT (9). Lastly, this review will discuss improvements in Treg production related to tissue source, Treg subsets, suppressor potency and stability, and potential for use as an off-the-shelf product capable of treating multiple recipients.

## CD4 Treg background

CD4+ Tregs can be divided into three main classes based upon site of development. Thymic Tregs (tTregs) are CD4+25++FoxP3+ cells formed in the thymus. Peripheral Tregs (pTregs)and induced Tregs (iTregs) acquire Foxp3 and suppressor function *in vivo* or *in vitro*, respectively. Type 1 regulatory T (Tr1) cells also can arise *in vivo* in the periphery or induced *in vitro*; Tr1 cells do not express FoxP3, require the transcription factors Tbet and B lymphocyte-induced maturation protein-1 (Blimp-1) (10), and secrete IL-10 as the primary mechanism for their suppressive function (11).

Regulatory CD4+ T cells (Tregs) are a rare cell type crucial for immune system homeostasis, limiting the activation and differentiation of effector T cells (Teff) that are self-reactive or stimulated by foreign antigen exposure (3). Treg are characteristically defined by the constitutive expression of both Foxp3 and high expression of CD25, compared to conventional T-cells (Tcon) which typically express significantly lower levels of both CD25 and Foxp3 (12). However, human Tcon can also transiently express Foxp3 following TCR stimulation, thus human FoxP3^+^ T-cells consist of a heterogeneous population of both Treg and activated Tcon. CD127 expression has been shown to inversely correlate with the expression of Foxp3 in human T-cells (13). Therefore, human Treg are characterized as CD127^lo^ (i.e. CD4^+^CD25^+^CD127^lo^Foxp3^+^). CD4+25++ Treg also co-express high levels of several immunosuppressive functional markers, including CTLA-4, Lag3, TIGIT, Tim-3 and PD-1, which directly contribute to the critical suppressive function of this population (14–17), as well as CD39 and CD73 (18). Treg also constitutively express a number of co-stimulatory molecules, including 4-1BB, OX-40, TNFRII, TNFRSF25 (19). While expression is not restricted to Treg, Helios and neuropilin-1 expression have been shown to increase Treg stability *in vivo* (20–22).

Interestingly, several mechanisms used by Treg for suppression of Teff responses also help stabilize the Treg phenotype. For example, high expression of CD25 by tTreg and iTreg may preferentially facilitate IL-2 signaling to Treg and, *via* competition, diminish IL-2 signaling of Teffs (23). Similarly, multiple subsets of tTreg, iTreg and Tr1 cells produce the immunosuppressive cytokines TGFß and IL-10, which concomitantly promote Treg stability while limiting Teff activation and differentiation (11, 23, 24). Treg also secrete the immunosuppressive cytokine IL-35, which has recently been shown to induce infectious tolerance and/or T cell exhaustion (25, 26). Treg also use metabolic intermediates to suppress T cell activation, including extracellular production of adenosine through the concordant expression of CD39 and CD73 (27) and the direct transfer of the potent inhibitory second messenger cAMP to T cells (23). Treg expression of CTLA4 can induce DC expression of indoleamine 2,3- dioxygenase (IDO), which suppresses *via* depletion of tryptophan and commensurate production of kynurenines (28).

Treg can also directly induce T cell death by several pathways. Human Treg and Tr1 cells can directly lyse T cells *via* a perforin and granzyme A or B mechanism, respectively (29, 30). Alternatively, Treg can induce T cell apoptosis *via* a TRAIL-DR5 pathway or through expression of galectin-1 (31) or FasL (32).

## CD4 Treg ACT clinical trials

Despite strong evidence of the *in vivo* efficacy of Tregs in murine and xenogeneic models, the initiation of clinical trials was slowed due to difficulties in obtaining sufficient numbers of Tregs without contaminating effector T cells (Teffs) that may subvert Treg potency and stability (33). Another consideration was that supra-physiological murine Treg numbers can cause generalized immunosuppression (34, 35). GVHD, a frequent and severe complicating factor in allo-HSCT (6), represented a unique Treg application venue as the GVHD risk period has a defined onset that begins with the infusion of a known number of donor T cells that can be controlled by certain T cell:Treg ratios. Furthermore, the goal of immunosuppression is to control donor anti-host reactions until the highest risk period has passed, facilitating the development of operational tolerance.

One of the biggest hurdles to the development of a successful GVHD therapy is maintaining the therapeutic GVL effect. There has been concern in the field that Treg ACT would result in global immunosuppression, interfere with an effective GVL response, and potentially induce an aggressive autoimmunity (36). Further concerns included the possibility that infused Treg would convert to Teffs, thereby worsening GVHD. However, murine and xenogeneic experiments showed that Treg did not exacerbate GVHD (32, 37–40). Indeed, over 20 reports on Treg ACT clinical trials found that Treg did not exacerbate GVHD. There is the potential loss of a GVL response. While preclinical studies do not support this as a substantial risk, clinical outcome parameters for cancer recurrence are not sufficiently mature to reach a definitive conclusion.

Several groups have now reported Treg ACT acute GVHD (aGVHD) prevention data with variations including whether Treg were *in vitro* expanded or freshly isolated and directly infused, type and source of Treg, and Treg dose (**Table 1**). In first-in-human Treg infusions, Treg were flow-sort purified from the initial allo-HSCT donors, expanded *in vitro*, and then infused into patients with acute or chronic GVHD. Transient improvement for aGVHD and significant reduction in symptoms and immune suppressive drugs were seen (46). In initial Treg ACT studies for GVHD prophylaxis, donor Tregs bead-purified from peripheral blood (PB), no toxicities were seen; however, a limited number of Tregs prevented dose escalation over 5x10^6^/kg studies (41, 42). Efficacy was observed in patients receiving Tregs prior to Tcon infusions, allowing *in vivo* Treg expansion to occur in lymphopenic recipients, allowing for higher Treg : Tcon ratios (44). To achieve higher Treg cell doses, bead-purified Tregs were expanded *in vitro*, albeit with lower purity (Foxp3+CD127-) and suppressor function. Adding rapamycin that preferentially inhibits Tcon over Treg expansion (51–54) to bead purified Treg cultures increased purity and suppressor function, allowing assessment of the efficacy of donor Treg ACT on GVHD (NCT00725062). In other concurrent studies, Tregs were purified from umbilical cord blood (UCB); *in vitro* expansion was achieved with retention of high purity and suppressor function due to a relative lack of contaminating Tcons in UCB as compared to PB. The initial study showed modest reduction in aGVHD in recipients of third-party expanded UCB blood Tregs at a dose of 3x10^6^/kg (4). In a follow-up study employing a second round of Treg expansion, doses of up to100x10^6^/kg virtually eliminated aGVHD with a cumulative incidence of only 9% at 100 days (5).

**Table 1:** Completed clinical trials with results involving adoptive Treg therapy in GVHD (search date March 30, 2022).

Treg type	Study ID	Patients	HSC product	Cell Product	Dose	Outcomes	Center	Ref’s.
Fresh Treg	2012-002685-12	9	Not specified	Fresh PB CD4 Treg Up to 5×10^6^/kg	Fresh CD4 tTreg Up to 5×10^6^/kg × once	Safe; not designed for efficacy	University Hospital Regensburg, Germany	(41)
	01/08	28	Haploidentical	Fresh PB CD4 Tregs and Tcons	2×10^6^/kg - 4×10^6^/kg Treg and 0.5×10^6^/kg - 2×10^6^/kg Tcon	15% developed ≥ grade 2 aGVHD 5% developed relapse	University of Perugia, Italy	(42, 43)
	NCT01660607	24	TCD MRD/MUD	Fresh PB CD4 Tregs and Tcons	1×10^6^/kg - 3×10^6^/kg Treg and 1×10^5^/kg - 3×10^7^/kg Tcon	1st cohort: 40% ≥ grade 2 aGVHD 2^nd^ cohort: No GVHD (n = 7)	Stanford, USA	(44)
	NCT02423915	5	dUCBT, n = 2 PB MUD, n = 3	fresh UCB CD4 Treg ± Fucosylation	1.2×10^6^/kg	100% ≥ grade 2 aGVHD	MD Anderson, USA	(45)
Expanded Treg	NKEBN/458-310/2008	2	MRD	Expanded CD4 Treg	3 × 10^6^/kg in SR aGVHD	Reduced IST in cGVHD. Only transient improvement in aGVHD	Medical University of Gdańsk, Poland	(46)
	NCT00602693	23	dUCBT	Expanded UCB CD4 Treg	0.01-3×10^6^/kg Treg	43% ≥ grade 2 aGVHD (vs. 61% in hist. control)	University of Minnesota, USA	(4)
	NCT00602693	11	dUCBT	Expanded UCB CD4 Treg	3×10^6^-1×10^8^/kg Treg	9% developed ≥ grade 2 aGVHD	University of Minnesota, USA	(5)
	EK 206082008	5	Any	Expanded PB CD4 Treg	5×10^5^/kg – 4.4×10^6^/kg × once	Clinical response to SR-cGVHD in 2 pts. Stable disease in 3 pts	University Hospital Carl Gustav Carus, Germany	(47)
		3	Any	Expanded donor PB CD4 Treg	3×10^6^/kg Treg	Clinical response to SR-cGVHD in 3 pts.	Charité – Universitätsmedizin Berlin, Germany	(48)
iTreg	NCT01634217	14	MRD	Expanded PB CD4 iTregs	Up to 3×10^8^/kg	2^nd^ cohort: 20% ≥ grade 2 aGVHD	University of Minnesota, USA	(7)
Tr1	ALT-TEN	18	Haplo	Expanded IL-10 Tr1 DLI	1-3x10^5^ CD3C T cells/kg	Grade 3 GVHD in 1/5 pts with immune reconstitution. No GVHD in 7 pts without immune reconstitution	San Raffaele University, Italy	(49)
	NCT03198234		Any	Expanded T-allo10 cells	1-9x10^6^-T-allo10/kg	Tr1 cells detected up to 1 yr after HSCT. Cont. recruitment.	Stanford, USA	(50)

HSCT, hematopoietic stem cell transplantation; cGVHD, chronic GVHD; GVHD, graft-verus-host disease; aGVHD, acute GVHD; MRD, matched related donor; MUD, matched unrelated donor; TAC, tacrolimus; CSA, cyclosporin; Siro, sirolimus; IST, immunosuppressive therapy; SR GVHD, steroid-refractory GVHD; dUCBT, double umbilical cord blood transplant; MMF, mycophenolate mofetil; PB, peripheral blood; UCB, umbilical cord blood.

Protocols have been developed to induce regulatory function in PB CD4 non-Treg cells by expanding Tcons in the presence of anti-CD3 antibody, TGFβ and rapamycin (37). These iTregs were as suppressive *in vitro* and *in vivo* as pTregs. Because PB Tcons are far more abundant than Tregs, yields were as much as 50-fold higher than initial PB and UCB Treg clinical trials. Despite concerns for iTreg de-differentiation to Teffs (termed plasticity), iTregs given as GVHD prophylaxis were well-tolerated at doses of 300x10^6^/kg with no clinical or laboratory evidence of iTreg plasticity (7).

Tr1s, initially shown to mediate tolerance following allo-HSCT in severe combined immune deficiency patients, have desirable properties such as antigen specificity and a direct graft-vs-leukemia (GVL) effect against some tumors (55). Tr1 ACT was then used in a proof-of-concept study treating patients receiving allo-HSCT for hematological malignancies (49). Their *in vivo* suppressive role is can best be demonstrated in situations in which Tregs are present at low to negligible levels such as aGVHD, wherein Tr1 become the main Treg subset; conversely, under these conditions, Tr1 deficiency can lead to GVHD progression (11). Roncarolo, Bachetta and colleagues are conducting a dose-escalation study (1-9x10^6^/kg) with host allo-antigen driven Tr1 cells; preliminary analysis shows that therapy is well-tolerated, with long-term persistence of Tr1 cells (50, 56).

In addition to the varied types of Treg used for ACT, these products differed in their state of differentiation. Most Treg ACT trials have used cells purified from PB as a readily accessible Treg cell source. The majority (>80%) of PB Tregs (and Tcons) are antigen-experienced (i.e. CD45RO+) and have been shown to expand to a lesser extent than their naïve counterparts (57–59). In contrast, Tregs isolated from UCB are >90% naïve (33) as are tTregs isolated from pediatric thymi often removed to better expose the operating field in children born with congenital heart defects (60, 61).

## Impact of different immunosuppressive drugs on Treg function

One significant consideration for the use of Treg ACT for either prophylaxis or treatment of GVHD are the wide range of immunosuppressants used in the transplant setting. Studies with murine and human T cells have shown that treatment with JAK inhibitors (Ruxolitinib, JAK1/2 or Pacritinib, JAK2) can increase the relative proportion of Treg following transplant (62, 63). Similarly, Treg expression of aldehyde dehydrogenase preferentially allows Treg compared to Teffector (Teff) survival in the presence of cyclophosphamide treatment during HSCT (64). Rapamycin, an mTOR inhibitor, allows preferential survival of Treg over Teff *in vitro* and *in vivo* (40, 52, 53), owing to Foxp3-mediated expression of Pim2, a kinase with substrate overlap with Akt and, by extension, mTOR (51). In contrast, cyclosporin A (CsA) inhibits Treg persistence and suppressor function *in vitro* and in an ACT model *in vivo* (65).

## Genetic engineering to improve Treg specificity and suppressor function

In preclinical studies, antigen-specific Tregs have superior potency on a per cell basis as compared to polyclonal Tregs and as a result of antigen-specificity, decreased risk of global immunosuppression (66–68). Although alloantigen-reactive Tregs can be expanded *via* repetitive stimulation with host antigen-presenting cells (APCs), clinical translation has proven to be challenging due to the low frequency of such tTregs and pTregs present in PB (69, 70).

To confer antigen specificity, polyclonal Tregs can be transduced with a recombinant antigen-specific TCR or CAR directed to the desired antigen (71–73). TCR delivery has been tested in various preclinical models of autoimmune diseases and transplantation (74–77). In the context of GVHD, Semple et al. showed that iTregs generated from chicken ovalbumin (OVA)-reactive CD4 OT-II TCR transgenic T cells efficiently prevented aGVHD induced by polyclonal Teffs in allogeneic recipients that expressed OVA protein, but not in OVA(-) recipients (78). In a subsequent study, Li et al. generated iTregs reactive to minor histocompatibility antigens that are encoded on the Y-chromosome. Male histocompatibility (H-Y)-specific iTregs isolated from TCR transgenic mice were highly effective in controlling GVHD in an antigen-dependent manner while sparing the GVL effect against acute or pre-established leukemia (79). While these studies provide a rationale for further development of TCR-specific Treg therapies, translating TCR gene modifications into the clinic for use in GVHD prophylaxis and treatment is hampered by the necessity that the host target antigens need to be presented in the context of a specific HLA determinant, or of the direct allorecognition of the “foreign” host HLA-determinant itself. Furthermore, mispairing of the endogenous and engineered TCR chains can cause undesired reactivity and off-target effects (80). Various strategies have been explored to reduce this issue, including genome editing techniques to partially knockdown or knockout endogenous TCR expression, as well as using TCR chains that are structurally modified in the constant region, such that they pair with endogenous chains with lower efficiency (81–83).

While TCRs can recognize both intracellular and surface antigens, CAR recognition is limited to cell surface proteins. However, CARs have the advantage of being MHC independent and their function can further be regulated *via* co-stimulatory signal potentiation (84, 85). Furthermore, Tregs possess a unique feature of bystander suppression which enables targeting of third-party antigens present in the same tissue to induce endogenous tolerogenic cells through a process known as infectious tolerance (86–89). This modality is particularly advantageous in diseases with no defined causative antigen (**Figure 1**).

The first CAR Tregs developed with the specific aim of reducing alloimmunity were targeted against HLA-A2, a frequently mismatched antigen in allo-HSCT (90). Tregs expressing an HLA-A2 CAR were shown to inhibit xenogeneic GvHD more effectively than polyclonal Tregs on a per cell basis (90). In subsequent studies, HLA-A2 CAR Tregs were shown to migrate to HLA-A2 expressing skin and islet grafts, alleviating the alloimmune-mediated graft rejection in humanized mice (91, 92). These promising results have led to the authorization of the first CAR-Treg clinical trial in the UK and the Netherlands (STeadfast) to evaluate the safety and tolerability of an autologous HLA-A2-specific Treg therapy (TX200-TR101 product) for HLA-A2 mismatched kidney transplant recipients (EUCTR2019-001730-34-NL and NCT04817774). Results of the STeadfast trial,may further support the application of CAR Tregs in a clinical trial setting, further expanding the possibility of using CAR Tregs in other disease conditions. As such, the results of this study are highly anticipated.

Another antigen recently applied to CAR Tregs for preventing GVHD in preclinical studies is CD19 expressed on B cells (85, 93). Using a xenogeneic GVHD model, Imura et al. showed that GvHD-suppressing effect of human CD19-CAR Tregs was greater than that of polyclonal Tregs in immune deficient mice given peripheral blood mononuclear cells, probably because such Tregs could specifically expand in response to B cells (93). As such, CD19-CAR Tregs may also be a potential candidate for treating chronic GVHD and antibody-mediated autoimmune conditions due to their capacity to inhibit antibody production (93).Several studies have investigated the effects of incorporating different costimulatory motifs into CAR Tregs. Dawson et al, compared 10 costimulatory domains, including CD28, 4-1BB, ICOS, CTLA-4, PD-1, GITR, OX40 and TNFR2, in a xenogeneic GVHD model using the HLA-A2 CAR Treg platform (85). These data, as well as those of three other independent studies, confirmed that CAR Tregs encoding a CD28 signal have superior *in vitro* and *in vivo* suppressor function (85, 93–95). These studies highlight the fact that intracellular signaling domains most effective in CAR-T cells do not necessarily apply to CAR-Tregs. Understanding how different CAR designs affect Treg function merits further exploration (71).

**Figure 2:**
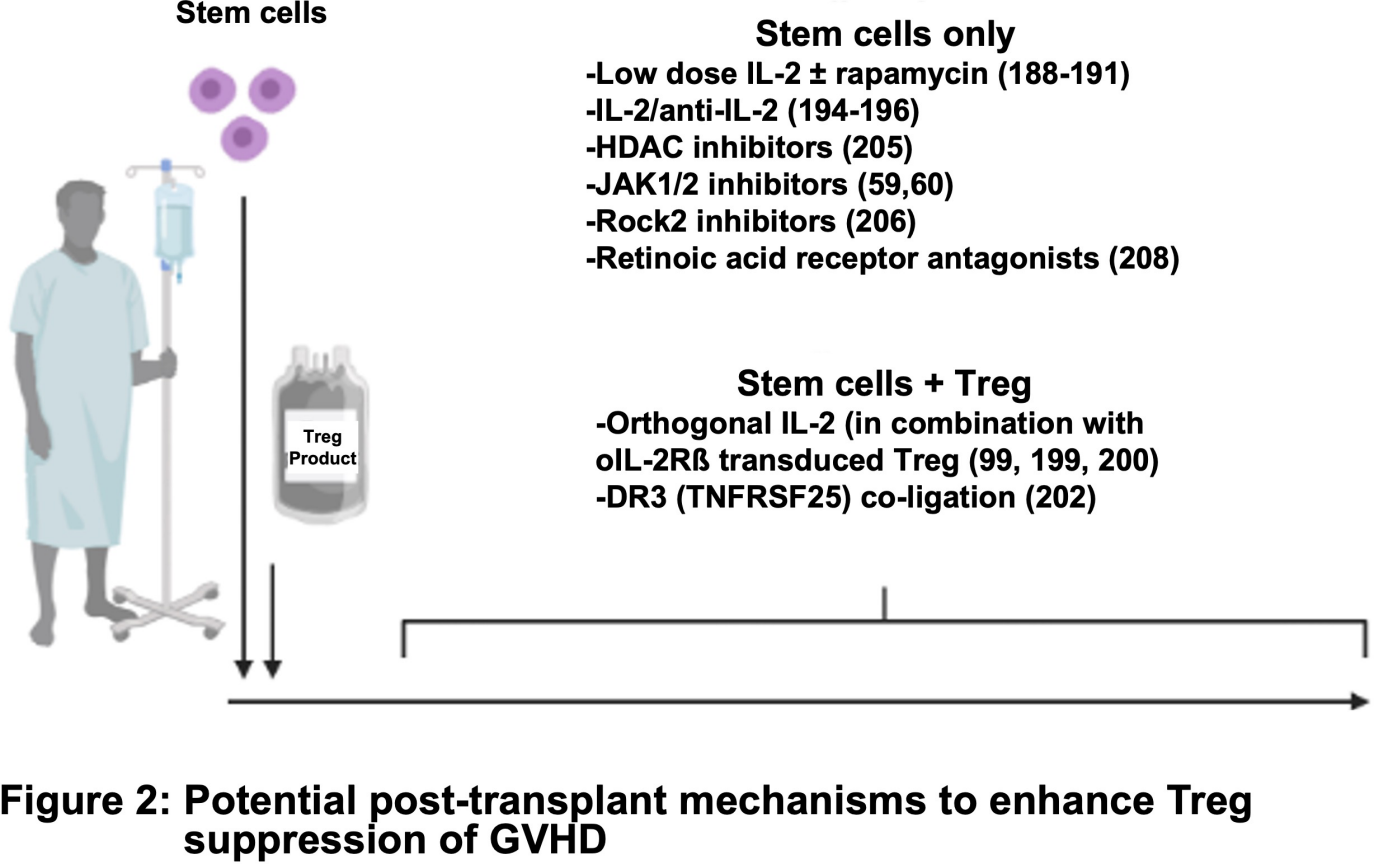
Potential post-transplant mechanisms to enhance Treg suppression of GVHD.

Recent advances in the field of cancer immunotherapy have inspired the adoption of innovative CAR designs. Rana et al. compared the functionality of a FVIII-specific second-generation CAR Treg with that of a TCR fusion construct (TruC) generated *via* linking of the FVIII scFV to CD3ϵ TCR chain (96). High-affinity second-generation CAR engagement led to strong TCR independent signaling and loss of Treg suppressor function along with limited *in vivo* persistence. In contrast, TruC Tregs delivered controlled antigen-specific, TCR-dependent signaling *via* engagement of the CAR along with the TCR complex to suppress FVIII-specific antibody response (96). Modular CARs, also known as universal CARs or switchable CARs, have also been applied to the field of CAR Tregs (97, 98). In this approach, the target antigen is not recognized directly by the CAR but rather by an adaptor encoding a tag such as biotin or fluorescein isothiocyanate (FITC) that is recognized by the CAR. A single CAR can thus be used to recognize a wide range of target antigens *via* a designated FITC- or biotin-conjugated antibody (97, 98). More recently, third generation CARs with two costimulatory motifs and fourth generation CARs which co-express constitutive or inducible factors such as cytokines or transcription factors have been developed (99–101). These have not been reported for Tregs to date; however, one can envision that a similar approach can be used to engineer a fourth generation CAR Treg with tailored cytokine support in order to modulate their function and stability more precisely (**Figure 2**) (102).

## FoxP3 gene editing to generate Tregs

Because of the challenges associated with isolating a pure population of Tregs, genetic engineering has been used to enforce FoxP3 expression (103, 104). Although initial studies showed that ectopic expression of FoxP3 could induce a regulatory phenotype, subsequent studies have shown that FoxP3 expression alone is not sufficient to imprint a stable (resistant to plasticity) and fully functional Treg phenotype (105–107). The difference between tTregs, pTregs and FoxP3-converted T cells may lie in the FoxP3 expression level needed to stabilize the Treg phenotype (106). Allan et al. highlighted the importance of delivering the FoxP3 gene with a strong promoter to drive constitutive expression with limited fluctuation depending on the cell activation state (105). Similar findings were reported by Honaker et al, who used DNA editing techniques together with a homology directed repair to insert a strong promoter into the endogenous *FOXP3* locus (108). More recently, Lam et al. published an optimized method for efficient and stable human Treg expansion with CRISPR-mediated *FoxP3* gene knock-in (109). Collectively, these efforts highlight the importance of novel directed gene editing techniques in the design and development of next-generation Treg therapies.

## Tissue targeting

It is well-established that Tregs found within different tissue niches can represent phenotypically and functionally distinct Treg subsets critical for local immune homeostasis and regulation of tissue-specific inflammatory disease, including GVHD (110–112). Treg heterogeneity is directly influenced by the immense diversity of cellular and non-cellular mediators in each specialized tissue microenvironment (110, 113, 114). As such, tissue niche-specific Treg subsets often have differential gene expression, including cytokine receptors that can provide a selective advantage within each tissue microenvironment (112, 115). Further, the mechanisms by which Treg migrate and infiltrate into these peripheral tissues have also been shown to play a critical role in immune regulation. Therefore, ex vivo Treg manipulation to facilitate homing to and survival within these tissue-specific niches may enhance the efficacy of Tregs *in vivo* in controlling those local environments.

Organ systems often take advantage of local tissue-specific stimuli to modulate local immune responses. In particular, tissue-specific Tregs are readily influenced by diverse environmental mediators within each distinct tissue microenvironment which may directly contribute to local immune homeostasis and the pathology of a wide-range of human disease, including GVHD (110–112, 116–119). For example, While.bone marrow (BM)-Tregs have several distinct characteristics and functional requirements that differ from other peripheral Treg populations, including differential upregulation of cytokine and chemokine receptors that may provide BM-Tregs with a unique selective advantage in that compartment (112). The BM niche is an extremely diverse and complex tissue (120–122). Previous work has suggested that the variable distribution and composition of different niches even within the BM itself can differentially impact important T-cell functions including proliferation, differentiation, migration and quiescence (112, 123). Similarly, unlike splenic Tregs, BM-Tregs proved to be minimally responsive to exogenous IL-2 given *in vivo*; instead, recombinant IL-9 significantly increased BM-Treg frequency while having no impact on the frequency of splenic Tregs (112). IL-9 is required for optimal maintenance of Treg suppressor function (124, 125). We observed both an upregulated expression of IL-9R in BM-Treg as well as an enhanced capacity to respond to IL-9 both *in vitro* and *in vivo*. Collectively, these data suggest that differential cytokine signaling within the BM niche may provide a distinct survival and functional advantage for BM-Tregs.

Similarly, within the gastrointestinal (GI) tract, differential expression and release of local simulants have been shown to both induce the production of pTregs within the gut and help to promote Treg localization and retention within the GI tract (126–130). The release of environmental factors, including TGF-β and retinoic acid (RA), drives local pTreg differentiation in the gut tissue (131–136) by contributing to gut immune homeostasis even under inflammatory conditions (137–139). Interestingly, T-cell *in vitro* exposure to RA and TGF-β is also associated with the induction of gut tropism and enhances the expression of several gut-associated T-cell homing receptors (126, 128).

Lymphocyte migration is well-established as a fundamental mechanism for the maintenance of normal immune function and is integral in controlling the pathology of inflammatory disease (140–142). Within the context of GVHD, T-cell and Treg homing can influence the initiation, severity, and prevention of GVHD (139, 143–150). Tissue-specific pathology within GVHD target organs, including the skin, liver, and GI tract is illustrative of the significance of T-cell and Treg homing mechanisms in GVHD pathology (139, 149, 151). In response to local inflammation and associated tissue damage, homing receptor ligands and chemoattractant receptors are upregulated by injured stromal cells (142, 152), providing directional cues for Teff and Treg migration to inflamed tissue. Because GI tract injury and inflammation are major drivers of disease severity (139, 145, 153–155), targeted the specific targeting of Tregs to the GI tract may be highly advantageous in mitigating disease severity and improving outcomes. Beilhack et al. (149), demonstrated that allogeneic donor T-cells first expanded within secondary lymphoid organs (SLO) then migrated to GVHD target organs. Similarly, this group later reported that Tregs were able to colocalize with allogeneic donor T-cells during GVHD, initially expanding within SLOs then migrating into inflamed tissues (148). Inflammation caused by irradiation and GVHD-associated pathology provided crucial stimuli for early Treg migration to these sites of donor T cell localization, reducing allogeneic T-cell proliferation and activation *in vivo* (148). Several studies have reported an integral role for GI homing of T-cells for both the initiation and prevention of GVHD (143, 145, 147, 156), although these findings can vary depending upon the intensity of conditioning and the pathogenic mechanisms responsible for GVHD (156). T-cell homing the GI tract is facilitated by distinct tissue-specific mechanisms that attract T-cells to the small or large intestines (126, 157–161). These pathways are primarily regulated by the expression of CCR9, α4β7 and GPR-15 (126, 127, 142, 157, 162–165). In particular, the expression of CCR9 and integrin α4β7 are integral to T-cell trafficking during GVHD. In a 2006 study Waldman et al. (145) demonstrated that alloreactive donor T-cells from α4β7-/- transgenic mice had a reduced capacity to cause GVHD, with a corresponding reduction in T-cell infiltration and tissue injury in both the gut and liver. Similarly, a retrospective case study of 59 allo-HSCT patients demonstrated that α4β7 expression was significantly upregulated in memory and naïve T-cell populations and CCR9 in CD8+ memory T-cells in patients who subsequently developed intestinal GVHD (147), studies that led to the testing of anti-α4β7 blocking antibody to prevent and treat aGVHD in the clinic (166–168).

Likewise, the expression of GI tract homing receptors has also been found to play a central role in Treg efficacy during allogeneic HSCT. Engelhardt et al. (143) recently reported that allo-HSCT patients with higher frequencies α4β7+ Treg post-transplant saw a significant increase in Treg infiltration within the GI tract, and correspondingly a reduced organ-specific risk and reduced GVHD severity. Interestingly, this study also reported a distinct negative correlation between the expression of cutaneous leukocyte antigen (CLA) in allogenic T-cell and the associated risk and severity of GVHD of the skin (143). During GVHD, skin involvement is often one of the first and most commonly manifestations of disease, with skin involvement occurring in >80% of aGVHD patients (169, 170). Like GI tract involvement, aGVHD of the skin can significantly impact allo-HSCT patient morbidity. CLA mediates T-cell homing to the skin by interacting its ligand, E-selectin, which is highly expressed on the microvasculature structure within the skin (171–173). This, in combination with the co-expression of several chemokine receptors, including CCR4, CCR6, CCR8, and CCR10, drives T-cell migration towards epithelial surfaces including the skin and GI tract (142, 146, 171, 174, 175). Varona et al. (146) also demonstrated a correlation between CCR6 expression in MHC class II–mismatched T-cells and the associated risk of GVHD in both the skin and GI tract with a significant reduction in the incidence and severity of GVHD in allogenic recipients of CCR6-deficient T-cells. Together, these studies support the notion that tissue-targeted Treg therapy may be a novel approach for GVHD therapies.

This then raises the question of how we can harness tissue-specific homing mechanisms for clinical translation? Recently, Hoeppli et al. (176) described an ex vivo human Treg product tailored to mimic gut-homing primed Tregs. Here, they utilized ex vivo RA stimulation to induced CCR9 expression in human PB CD4+Foxp3+ Tregs (176) and demonstrated that the ex vivo induction of CCR9 expression was sufficient to enhance Treg migration to the GI tract and reduce disease severity in a xenogeneic GVHD model (176). GPR-15 expression, an understudied chemoattractant homing receptor (127, 143, 176), has been shown to be highly dependent on environmental stimuli and regulated by TGF-β within the GI tract (127, 128) and an environmental chemical sensor, aryl hydrocarbon receptor (AHR) (177, 178). The ligand of GPR-15, GPR-15L, has been reported to be highly expressed in epithelial tissues exposed to the environment, including the skin and GI tract (179, 180). Together, these data suggest that GPR-15 is another promising target for a targeted Treg therapy. In addition to the ex vivo induction of tissue-targeted Treg products, genome modification of Tregs to achieve ectopic expression of T-cell homing receptors The generation of tailored tissue-targeted Tregs has the potential to increase the targeted efficacy of Tregs *in vivo* while reducing the risk of more global immunosuppression by providing a selective advantage for targeted Treg products.

## Enhancing ex vivo Treg expansion and stability

As discussed earlier, rapamycin improves both culture purity and suppressor function for clinical Treg ACT. A platform has been developed for solid organ transplant in which allo(donor)-specific Tregs from healthy donors or recipients post-transplantation are expanded in the presence of co-stimulatory blockade. Such Tregs maintain *Foxp3* demethylation status which strongly correlates with stability (181, 182).

Expansion of sort-purified human Treg in the presence of TNFα and IL-6 increases expansion ~3-fold while maintaining Foxp3 expression, demethylation status, and *in vitro* and *in vivo* suppressive function (183). PKC-Ø is a negative regulator of Treg suppressive function, and acute treatment of expanded Treg with a non-competitive PKC-Ø inhibitor (AEB071) increased *in vitro* and *in vivo* suppressor function (184). Downregulation of miR-146b, which targets Traf6, increased Treg suppressive function *in vitro* and GVHD efficacy *in vivo* (185). Following *in vitro* expansion, purified CD39hi vs CD39lo Tregs were more suppressive in a xenogeneic GVHD model (186). Adoptive transfer of IL-33 stimulated Tregs were more effective than control Tregs at preventing murine aGVHD (187) an effect dependent on Treg expression of amphiregulin that can mediate tissue repair. In response to IL-33, engineered human ST2 (IL-33R)-expressing Tregs had increased expansion, maintained suppressor function, produced amphiregulin and had a heightened ability to induce anti-inflammatory M2 macrophages (188). IL-27, a member of the IL-12 family, has been shown to increase tTreg suppressive function and aGVHD efficacy in murine studies. Acute IL-27 stimulation increased the *in vitro* and *in vivo* suppressive function of human iTregs in a xenogeneic GVHD model (189). Lastly, CD155+ (DNAM+) Treg were less stable; depleting these cells at the beginning of culture increased Foxp3 expression, demethylation, and suppressive function *in vitro* (190).

## *In vivo* strategies to enhance Treg efficacy

Tregs have high expression of CD25 (the high-affinity subunit of the IL-2 receptor) and IL-2 is required for stability and expansion. Clinical trials have shown that prophylactic administration of low doses of IL-2 can expand graft-associated Tregs after allo-HSCT and reduce the incidence of acute and chronic GVHD (191–193). Low dose IL-2/rapamycin enhanced the long-term persistence of adoptively transferred Tregs in non-human primates in a non-GVHD setting (194). PEGylation of IL-2 was found to increase half-life *in vivo* and expand Tregs in a xenogeneic GVHD model (195). In other studies, murine and human Treg containing IL-2 nanogel ‘backpacks’ that deliver IL-2 to Tregs in an autocrine fashion under certain conditions that trigger the TCR at sites of antigen encounter showed increased suppression of skin graft rejection in murine and xenogeneic models of disease (196). Infusion of IL-2/anti-IL-2 complexes increased both IL-2 half-life and Treg numbers, along with suppressing murine diabetes, colitis, and skin allograft rejection (197–199). One group also showed that IL-2/anti-IL-2 could reduce disease in a xenogeneic GVHD model, although efficacy in the context of Treg ACT was not assessed (199).

However, IL-2 also can stimulate CD8 T cells and NK cells that express the high affinity IL-2 receptor. Exogenous low-dose IL-2 and IL-2/anti-IL2 complexes decreased Treg efficacy when given at the time of donor T-cell infusion in either xenogeneic or allogeneic GVHD models, respectively, likely though expansion of contaminating cells (200, 201). To circumvent IL-2 augmentation of CD8 T-cells and NK cells, the Garcia group engineered orthogonal IL-2/IL-2Rβ pairs for murine and human systems. Following introduction of an ortho-IL-2Rß subunit and administration of ortho-IL2 protein into murine and human T cells, these neo-cytokines increased *in vivo* tumor killing in T cell, and CAR T cell, ACT (102, 202, 203). Infusion of ortho-IL-2 protein that has a markedly reduced capacity to bind to cells expressing wildtype IL-2Rβ, with Tregs transduced to express the ortho-IL-2Rß subunit was effective in ameliorating murine heart allograft rejection (204).

*In vivo* Treg expansion and suppression of GVHD were augmented by stimulation through TNFRSF25 (DR3) with either an agonistic antibody or a form of the natural ligand (TL1A-Ig) (205). These *in vivo* expanded Tregs also had increased efficacy following adoptive transfer (206). Activation of TNFRSF-member (TNFR2) expanded Tregs *in vivo* and ameliorated GVHD, without the need for exogenous IL-2 (207). Additionally, several pharmacologic agents favor Treg over Teff cell expansion post-HSCT, including: histone deacetylase inhibitors (vorinostat), hypomethylating agents (decitabine), JAK1/2 inhibitors (Ruxolitinib), ROCK1/2 inhibitors (Belumosudil) (62, 169, 208–210) and RA receptor agonists (211).

## Concluding remarks

Treg ACT for GVHD prevention is now a reality, although barriers remain to common clinical practice. Pre-clinical advances are being made to enhance Treg efficacy, specificity, and tissue targeting. The clinical efficacy of adoptive Treg therapy for aGVHD is still being optimized. Comparable to the confluence in timing between Treg persistence and the relatively short-term immunosuppression needed in allo-HSCT and the fact that third party Treg ACT suppresses GVHD makes possible the production of banked (stored) Tregs that could be used to treat a multitude of patients. Importantly, Treg can also be highly expanded *in vitro* without obvious signs of exhaustion (212), enabling many of the culture or genetic manipulations discussed herein. Since Treg cryopreservation, an intricate part of banking, has proven very challenging (38, 61, 213), with varying recoveries, effects on Foxp3 expression, and *in vitro* suppressive functions, cryopreservation and thawing parameters that maintain a Treg phenotype and *in vivo* suppressive function after thawing is key to fully unlocking Treg ACT for GVHD and other indications such as graft rejection and autoimmune disease.

## Author contributions

All authors listed have made a substantial, direct and intellectual contribution to the work, and approved it for publication.

## Funding

This work was supported by grants from the Children’s Cancer Research Fund and National Institutes of Health, National Heart, Lung and Blood Institute grant and R01 HL114512-01 (K.L.H.), and R01 HL11879 and HL155114, National Cancer Institute grants P01 CA142106 and P01 CA065493, National Institute of Allergy and Infectious Diseases grants P01 AI056299, and R37 AI344495 (B.R.B.).

## Conflict of interest

The authors declare that the research was conducted in the absence of any commercial or financial relationships that could be construed as a potential conflict of interest.

## Publisher’s note

All claims expressed in this article are solely those of the authors and do not necessarily represent those of their affiliated organizations, or those of the publisher, the editors and the reviewers. Any product that may be evaluated in this article, or claim that may be made by its manufacturer, is not guaranteed or endorsed by the publisher.

## Abbreviations

ACT, Adoptive cell therapy; AHR, aryl hydrocarbon receptor; allo-HSCT, allogeneic hematopoietic stem cell transplantation; APCs, antigen-presenting cells; Blimp-1, B lymphocyte-induced maturation protein-1; BM-Treg, bone marrow Treg; CLA, cutaneous leukocyte antigen; Tcons, conventional T cells; Teff, effector T cells; FITC, fluorescein isothiocyanate; GI, gastrointestinal; GVL, graft-vs-leukemia; GVHD, graft versus host disease; iTregs, induced Treg; PB, peripheral blood; pTregs, peripheral Tregs; SLO, secondary lymphoid organs; Tregs, regulatory T cells; TCR, T cell receptor; TruC, TCR fusion construct; tTregs, Thymic Tregs; Tr1, type 1 regulatory T cells.
